# Better clinical outcomes and lower triggering of inflammatory cytokines for allogeneic hematopoietic cell transplant recipients treated in home care versus hospital isolation – the Karolinska experience

**DOI:** 10.3389/fimmu.2024.1384137

**Published:** 2024-08-07

**Authors:** Olle Ringdén, Britt-Marie Svahn, Guido Moll, Behnam Sadeghi

**Affiliations:** ^1^ Translational Cell Therapy Research, Division of Pediatrics, Department of Clinical Science, Intervention and Technology (CLINTEC), Karolinska Institutet, Stockholm, Sweden; ^2^ BIH Center for Regenerative Therapies (BCRT), Charité Universitätsmedizin Berlin, Berlin, Germany; ^3^ Julius Wolff Institute (JWI), Charité Universitätsmedizin Berlin, Berlin, Germany; ^4^ Department of Nephrology and Internal Intensive Care Medicine, all Charité Universitätsmedizin Berlin, corporate member of Freie Universität Berlin, Humboldt-Universität zu Berlin, and Berlin Institute of Health (BIH), Berlin, Germany

**Keywords:** allogeneic hematopoietic cell transplantation (alloHCT), graft-versus-host disease (GVHD), steroid-resistant GvHD, inflammation, cytokines, home care, oral nutrition, morbidity and mortality

## Abstract

After allogeneic hematopoietic cell transplantation (Allo-HCT) and conditioning, patients are typically placed in isolated hospital rooms to prevent neutropenic infections. Since 1998, we’ve offered an alternative: home care for patients living within a one to two-hour drive of the hospital. In Sweden this approach includes daily visits by an experienced nurse and daily phone consultations with a unit physician. When necessary, patients receive transfusions, intravenous antibiotics, and total parenteral nutrition at home. Our initial study report compared 36 home care patients with 54 hospital-treated controls. Multivariate analysis found that home care patients were discharged earlier to outpatient clinics, required fewer days of total parenteral nutrition, had less acute graft-versus-host disease (GVHD) grade II-IV, and lower transplantation-related mortality (TRM) and lower costs. Long-term follow-up showed similar chronic GVHD and relapse rates in both groups, with improved survival rates in the home care group. A subsequent comparison of 146 home care patients with hospital-treated controls indicated that home care and longer home stays were associated with lower grades of acute GVHD. Home care was found to be safe and beneficial for children and adolescents. Over two decades, 252 patients received home care post-Allo-HCT without any fatalities at-home. Ten-year outcomes showed a 14% TRM and a 59% survival rate. In 2020, an independent center confirmed the reduced risk of acute GVHD grades II-IV for patients treated in home care. Here, we report for the first time that home care patients also demonstrate a less inflammatory systemic cytokine profile. We found higher levels of IFN-γ, IL-2, IL-5, IL-13, GM-CSF, and G-CSF, but lower VEGF in hospital-treated patients, which may contribute to acute GVHD grades II-IV. In conclusion, home-based treatment following Allo-HCT yields multiple promising clinical outcomes and improved systemic inflammatory markers, which may contribute to less development of life-threatening GVHD.

## 1 Introduction

Bacterial and fungal infections are common during the pancytopenic phase following allogeneic hematopoietic cell transplantation (Allo-HCT). They are a major risk for treatment related morbidity and mortality and should thus be avoided for optimal patient care.

In-hospital patient isolation practices typically include the use of Laminar Airflow (LAF) rooms or standard protective isolation in single rooms, sometimes with HEPA-filtered air. These rooms, combined with strict handwashing and the use of gloves, gowns, and masks by staff and visitors, aim to reduce any infection risks.

A prospective, randomized study comparing LAF room isolation with standard procedures found significantly fewer cases of septicemia and major local infections in the LAF group, although survival rates were not significantly different (1). Furthermore, a large multicenter study showed that patients in HEPA or LAF rooms had reduced transplantation-related mortality (TRM) and improved survival compared to those in standard isolation (2).

Outpatient chemotherapy has been administered to recipients of autologous hematopoietic cell transplantation (Auto-HCT) (3). In addition, some centers even allow patients to spend a few hours or the night at home (4).

When starting our home care program in the Stockholm region, we suggested that patients could benefit from receiving HCT-related home care instead of hospital isolation. This idea, supported by the hospital’s infection control department, was met with a mix of support and skepticism among our peers. However, the Swedish Cancer Society provided financial backing for this project to systematically study the impact of home care versus hospital isolation in the HCT setting.

Consequently, in 1998, we launched a first systematic program offering home care to patients living within a 1-2-hour drive radius of Huddinge Hospital as an alternative to traditional isolation, featuring reversed isolation and HEPA-filtered air.

Outpatient Auto-HCT is well-established (5–7). Patients receiving non-myeloablative conditioning and Allo-HCT were also followed in outpatient settings (8–10). McDiarmid and colleagues reported that auto-HCT and Allo-HCT patients followed in the outpatient clinic post-transplant had fewer infections compared to those isolated in the hospital (11). They concluded that inpatient and outpatient Auto-HCT and Allo-HCT had similar outcomes. Similarly, Solomon et al. reached the same conclusion in an outpatient Allo-HCT study (12). Russell and colleagues opted for a hybrid model, providing a hospital bed for the initial two weeks post Allo-HCT but allowing patients to return home without changing their home environment (13).

Outpatient approaches require frequent, sometimes daily, hospital visits for examinations and blood tests at the outpatient clinic. With outpatient HCT, patients spend time regularly visiting the hospital. In contrast, our home care model allows patients to stay home, with nurses visiting for check-ups, blood sampling, injections, and transfusions as needed. Conditioning, whether myeloablative or reduced intensity (RIC), is administered in the hospital, and following graft infusion, the patients return home. Physicians provide afternoon calls or more frequent contact if necessary.

**Figure 1:**
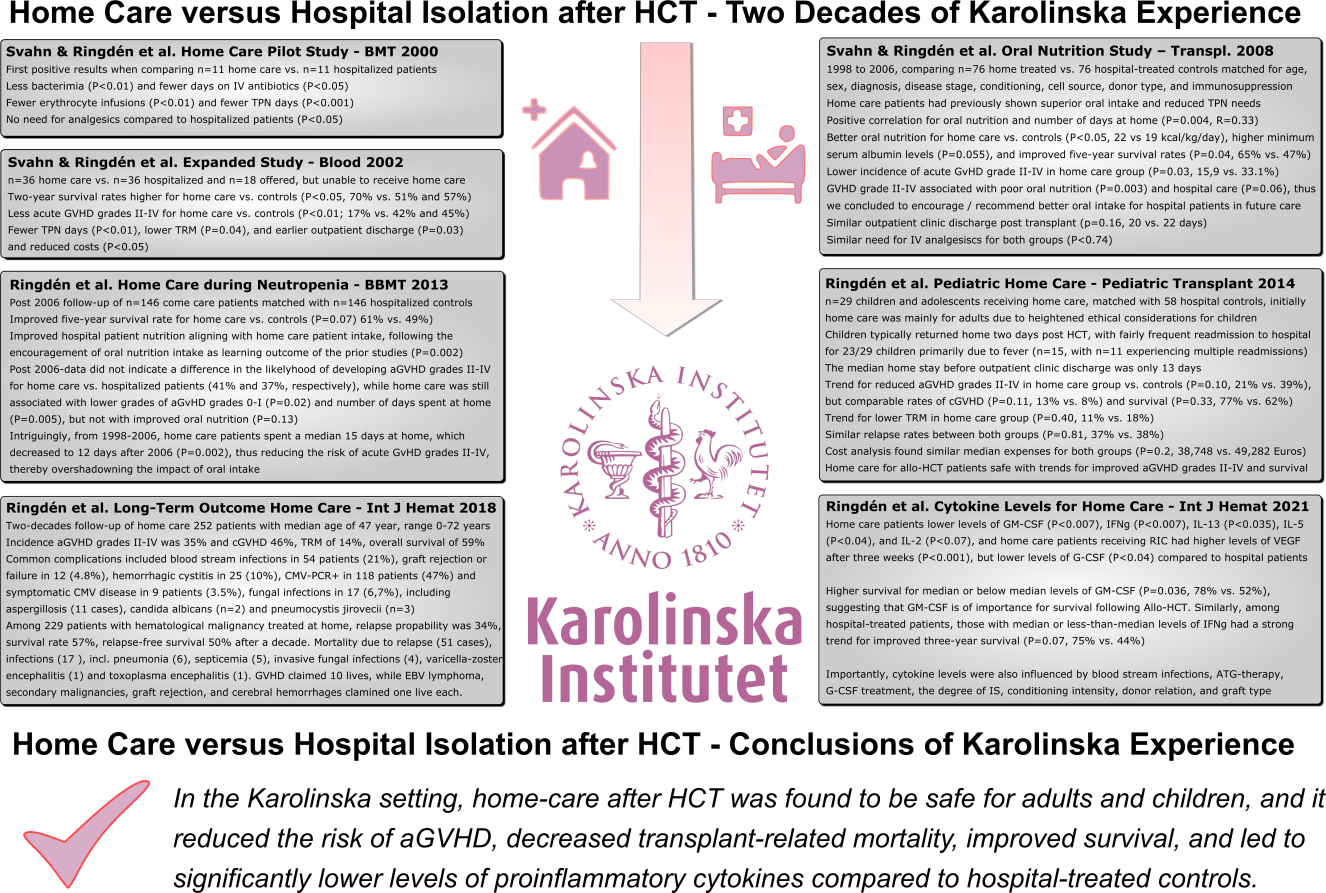
Home Care versus Hospital Isolation in HSCT – Two Decades of Karolinska Experience. This figure summarizes the major achievements made during the past two decades of home care post Allo-HSCT in this innovative care setting, illustrating the subsequent iterative steps from the conceptional idea and first pilot study reported in 2000 to the final long-term outcomes presented in the past years.

This article synthesizes our two decades of experience made with patient home care compared to hospital isolation post Allo-HCT in this innovative care setting, illustrating the subsequent iterative steps from the conceptional ideas and first pilot study reported in 2000 to the final long-term outcomes presented in the past years (**Figure 1**).

## 2 Home care study long-term follow-up and adjunct results

### 2.1 Patients receiving home care have decreased risk of moderate to severe acute GVHD

Our initial pilot study assessed the safety of providing home care after Allo-HCT (14). Patients living within a one to two-hour drive from the hospital were eligible for at-home treatment. A requirement for this option was having a caregiver—either a relative or friend—available to assist the patient. Additionally, the home environment had to be approved by the Department of Infection Control, which required water temperatures above 50°C, the absence of potted plants and pets, thrice-weekly bedding changes, and weekly cleaning.

Following unit-based conditioning and graft reception, patients were allowed to return home. There, an experienced nurse conducted daily visits to monitor the patient’s health, perform blood draws from central lines, and administer intravenous platelet or erythrocyte transfusions, total parenteral nutrition (TPN), and antibiotics when necessary, replicating hospital procedures. A physician reviewed test results daily, consulted with the home-care nurse, and contacted the patient as needed.

Of the 22 patients offered home care in the pilot, 11 accepted and met the inclusion criteria, while the others served as controls (14). In the home care cohort, three patients developed bacteremia compared to nine in the control group (p<0.01). Home care patients also had fewer TPN days (median 3 vs. 24, p<0.001), required fewer erythrocyte transfusions (median 4 vs. 8, p<0.01), and spent fewer days on intravenous antibiotics (median 6 vs. 13 days, p<0.05), with no need for analgesics (p<0.05) compared to hospitalized controls. These findings led us to conclude that home care was not only safe but also superior to hospital isolation.

The home care initiative continued, comparing 36 home care patients with two control groups: 18 patients who were offered but unable to receive home care for various reasons and 36 patients from other regions treated in the hospital (15). The home care patients typically returned home one day after graft infusion. Two were too ill to leave the hospital, and of the 34 treated at home, 21 were readmitted on 33 occasions (median 1 day, range 0-25 days), primarily due to fever, spending a median of 16 days (range 0-26 days) at home.

Multivariate analysis revealed several benefits of home care, including earlier outpatient clinic discharge (p=0.03), fewer TPN days (p<0.01), less acute GVHD grades II-IV (p<0.01), lower TRM (p=0.04), and reduced costs (p<0.05, **Table 1**). The incidence of grades II-IV acute GVHD was 17% among home care patients, significantly lower than the 42% and 45% observed in the control groups (p<0.05). Moreover, two-year survival rates were higher in the home care group (70%) compared to the control groups (51% and 57%, respectively, p<0.05). Thus, treatment at home, rather than hospital isolation, offered many advantages, most notably a reduced risk of developing acute GVHD grades II-IV.

**Table 1:** Improvements through home care compared to hospital care in allogeneic hematopoietic cell transplantation.

Univariate analysis			
	Home Care	Hospital Isolation	P-value
Days to discharge, Median	19	26	<0.01
Days with TPN, Median	4	14	<0.01
Acute GVHD II-IV	17%	44%	<0.05
TRM	8%	38%	<0.01
Costs until discharge, USD	25346	32226	<0.001
Multivariate analysis			
Factors	RR	C.I.	P-value
Days of discharge	0.33	0.12 –0.90	0.03
Days of TPN	0.24	0.09 –0.64	<0.01
Acute GVHD II-IV	0.25	0.09 –0.75	0.01
TRM	0.22	0.05 –0.96	0.04
Costs hospital care	2.7	1.01 –7.14	<0.05

From Svahn, BM et al, Blood 2002; 100:4317-4324.

RR, Relative Risk; C.I., Confidence Interval; TPN, total parenteral nutrition; GVHD, graft-versus-host disease; TRM, transplantation-related mortality.

Univariate and Multivariate analysis for outcomes.

### 2.2 Impact of home-based care on post-transplant outcomes: a comparative study of oral nutrition and acute GVHD incidence

Between 1998 and 2006, 601 patients underwent Allo-HCT at the Center for Allogeneic Stem Cell Transplantation (CAST), Karolinska University Hospital, Huddinge. Of these, 76 were treated at home and compared to a matched control group of 76 hospital-treated patients, matched by age, sex, diagnosis, disease stage, conditioning, cell source, donor type, and immunosuppression (16). This study emphasized oral nutrition, an area where home care patients had previously shown superior oral intake and reduced TPN needs compared to their hospital counterparts (14, 15). Median oral caloric intake was calculated in kcal/kg/day over the first 21 days post-HCT.

Home-treated patients had a median of 20 days (range 10-114) to outpatient clinic discharge post-transplant, compared to 22 days (range 8-104) for the hospital group (p=0.16). The median number of days requiring IV analgesics was zero for both groups, home-treated and hospital controls (0-87 vs. 0-72, p=0.74). Nonetheless, home-treated patients achieved better oral nutrition, with a median intake of 22 (range 4-48) kcal/kg/day versus 19 (range 0-43) kcal/kg/day in the control group (p<0.05) and recorded higher minimum serum albumin levels (p=0.055). A positive correlation was found between the number of days at home and oral nutrition (r=0.33, p=0.004). Additionally, the incidence of acute GVHD grades II-IV was lower in the home care group at 15.9% compared to 33.1% in hospital controls (p=0.03), with five-year survival rates at 65% versus 47%, respectively (p=0.04).

Multivariate analysis showed acute GVHD grades II-IV was associated with poor oral nutrition (p=0.003) and hospital care (p=0.06). Improved oral nutrition was believed to contribute to the lower risk of acute GVHD grades II-IV in home-treated patients (16). Following these findings, we have since encouraged better oral intake among hospital-treated patients, including a dedicated nursing effort to promote improved dietary consumption.

A subsequent study of 146 home care patients, matched with an equal number of hospital controls (17), showed that intensified oral intake efforts since 2006 led to improved hospital patient nutrition (p=0.002), aligning with home care patient intake levels. However, post-2006 data revealed no significant difference in the likelihood of developing acute GVHD grades II-IV between the two groups, with rates of 41% for home care patients and 37% for hospital patients. From 1998 to 2006, home care patients spent a median of 15 days at home, which decreased to 12 days after 2006 (p=0.002).

Multivariate analysis indicated that lower grades of acute GVHD (grades 0-I) were associated with home care (HR 41, p=0.02) and the number of days spent at home (HR 0.92, p=0.005), but not with oral nutrition (HR 0.98, p=0.13). The study also found a five-year survival rate of 61% in the home care group compared to 49% in hospital controls (p=0.07).

In conclusion, the duration of home care was a key factor in reducing the risk of acute GVHD grades II-IV, overshadowing the impact of oral intake.

### 2.3 Pediatric home care outcomes post-HCT

Initially, the home care project for Allo-HCT was exclusive to adults due to heightened ethical considerations in pediatric novel therapies. However, the program’s success led to requests from parents for home-based care for their children scheduled for Allo-HCT, prompting the extension of this option to pediatric patients.

Our study examined 29 children and adolescents receiving home care, matched with 58 hospital controls based on variables like age, diagnosis, disease stage, donor type, HLA compatibility, conditioning, and stem cell source (18). These children typically returned home two days post-HCT (range 1-15). In the home care group, 23 of the 29 children were readmitted to the hospital, primarily due to fever (n=15), with 11 experiencing multiple readmissions.

The median home stay before outpatient clinic discharge was 13 days (range 2-24). Acute GVHD grades II-IV occurred in 21% of the home care group versus 39% in hospital controls (p=0.10), with comparable rates of chronic GVHD at 13% and 8%, respectively (p=0.11). TRM was lower in the home care cohort at 11% compared to 18% among controls (p=0.40), and relapse rates were similar between groups (37% vs. 38%, p=0.81). Survival rates two to four years post-transplant were 77% in the home care group and 62% in hospital controls (p=0.33).

Cost analysis showed median expenses of 38,748 euros for home care patients versus 49,282 euros for hospital controls (p=0.20). The data suggested that home treatment for pediatric Allo-HCT patients was safe, with trends indicating potentially improved outcomes for acute GVHD grades II-IV and survival in the home-based care setting.

### 2.4 Long-term outcomes in home care post-HCT

After two decades of home care experience following Allo-HCT, we conducted an extensive follow-up (19). The cohort consisted of 252 patients with a median age of 47 (range 0-72). Myeloablative conditioning was administered to 102 patients, while 150 received reduced-intensity conditioning. The donor pool included 71 HLA-identical siblings, 160 matched unrelated donors, and 21 HLA mismatches.

Common complications included bloodstream infections in 54 patients (21%), graft rejection or failure in 12 (4.8%), and hemorrhagic cystitis in 25 (10%). Cytomegalovirus (CMV) PCR positivity occurred in 118 patients (47%), managed with antiviral medications (20). Symptomatic CMV disease was noted in nine patients and proven or probable invasive fungal infections occurred in 17 (6.7%), including aspergillosis (11 cases), Candida albicans (2), and Pneumocystis jirovecii (3).

The cumulative incidence of acute GVHD grades II-IV was 35%, with chronic GVHD at 46%. At the ten-year mark, TRM stood at 14%, with an overall survival rate of 59%. Among the 229 hematologic malignancy patients treated at home, the relapse probability was 34%, the survival rate was 57%, and the relapse-free survival rate was 50% after a decade.

Mortality was primarily due to relapse (51 cases) and infections (17 cases), including pneumonia (6), septicemia (5), invasive fungal infections (4), varicella-zoster encephalitis (1), and toxoplasma encephalitis (1). GVHD claimed ten lives, while Epstein-Barr virus lymphoma, secondary malignancies, graft rejection, and cerebral hemorrhages claimed one death each.

### 2.5 Cytokine kinetics and GVHD in allo-HCT: home care vs. hospital isolation

Serum cytokine levels in patients receiving home care following Allo-HCT and their hospital-isolated counterparts were assessed using the Luminex platform (**Table 2**) (21). The home care patients and hospital controls had weekly serum samples stored from the day of transplantation until discharge to the outpatient clinic.

**Table 2:** Cytokines decreased in multivariate analysis in home care patients in contrast to patients isolated in the hospital.

Cytokines	Week	Factor	RH	95% CI	P-value
GM-CSF	1	Home care	0.74	0.60 – 0.92	0.007
	2	GVHD II-IV	1.28	1.04 – 1.58	0.02
	2	Home care	0.77	0.63 – 0.95	0.02
IFN-γ	1	Home care	0.74	0.60 – 0.92	0.007
	2	Home care	0.77	0.62 – 0.96	0.02
	2	BSI	0.79	0.63 – 0.98	0.03
IL-13	1	Home care	0.79	0.63 – 0.98	0.035
	2	BSI	0.76	0.62 – 0.95	0.016
	2	Home care	0.82	0.66 – 1.01	0.06
IL-5	1	RIC	0.57	0.44 – 0.73	<0.001
	3	Home care	0.79	0.62 – 0.99	0.04
IL-2	1	Home care	0.81	0.65 – 0.01	0.07
	2	ATG	0.77	0.62 – 0.96	0.02

From O. Ringdén et al., International Journal of Hematology 2021; 113: 712 – 722.

RH, relative hazard; 95% CI, 95% Confidence interval; GVHD, graft-versus-host disease; BSI, blood stream infection; RIC, reduced intensity conditioning; ATG, anti-thymocyte globulin.

Furthermore, home care patients and hospital controls were matched for age, diagnosis, remission status, timing, HLA match, donor type (sibling or matched unrelated donor), and had similar numbers of female donors to male recipients, G-CSF treatment post Allo-HCT, donor age, CD34+ cell dose, and cell source (21).

Serum samples, collected weekly for the first three weeks post Allo-HCT, revealed that home care patients exhibited significantly lower levels of GM-CSF (p<0.007), IFN-γ (p<0.007), IL-13 (p<0.035), IL-5 (p<0.04), and IL-2 (p<0.07) (**Table 2**), altogether indicating less systemic inflammation in their cardiovascular system.

A separate analysis of patients receiving RIC showed that those in home care had higher levels of vascular endothelial growth factor (VEGF) after three weeks compared to hospital-isolated controls (p<0.001). Indeed, both, the patient intrinsic or the therapy-induced levels of VEGF in the vasculature may be indicative and of key importance for vascular integrity and vascular tissue healing in GVHD and hemorrhagic cystitis (22–28).

Importantly, survival was significantly higher (p=0.036) in patients with median or below-median levels of GM-CSF, being 78% at three years compared to 52% survival in patients with higher levels. This suggests that GM-CSF is crucial for survival following Allo-HCT and may be further somewhat indicative of bone marrow integrity and function (22).

In addition, among hospital-treated patients, those with median or less-than-median levels of the typical pro-inflammatory cytokine IFN-γ had a three-year survival of 75% compared to 44% for those with higher-than-median IFN-γ levels (p=0.07), indicating that less inflammation related to IFN-γ signaling may be beneficial, as also observed in other transplant settings (29).

Noteworthy, cytokine responses were influenced by multiple other confounding factors, including bloodstream infections, anti-thymocyte globulin (ATG) therapy, G-CSF treatment, the degree of immunosuppression, conditioning intensity, donor relation, and graft type.

In this cohort, 10% of home care patients experienced acute GVHD grades III-IV, compared to 16% among hospital controls. The cumulative incidence of acute GVHD grades II-IV was 42% and 49% in the two groups, respectively. The five-year survival rates were 69% for home care patients and 57% for those in hospital care.

In summary, hospital-treated patients showed increased levels of inflammatory cytokines implicated in the development of acute GVHD grades II-IV compared to those treated at home.

## 3 Discussion

The foundational objective of integrating home care into the HCT process was based on the idea that patients would benefit from the comfort and familiarity of their home environment rather than the isolation of hospital stays (**Figure 1**). This patient-centered approach not only aimed to enhance the quality of life during the treatment period but also aspired to foster a sense of normalcy during a challenging time (14, 15). The lower probability of acute GVHD grades II-IV, lower TRM, reduced costs, and other advantages with home care compared to hospital isolation were welcomed surprises (**Table 1**). The lower risk of acute GVHD grades II-IV in home care Allo-HCT was recently independently confirmed in a study from Barcelona (30).

There may be several reasons for a lower risk of acute GVHD grades II-IV at home compared to the hospital. One possibility is that patients are more adapted to the bacterial flora at home compared to that in the hospital. The hospital microenvironment may be more hostile and trigger inflammatory cytokines and acute GVHD grades II-IV more than the home environment (21). The microbiome is a predictor of outcome following HCT (31). In HCT patients, the intestinal microbiome is altered by a loss of diversity (32, 33). For instance, gnotobiotic mice have a decreased risk of developing GVHD (34). In a clinical study, patients with severe aplastic anemia undergoing HCT had a lower probability of acute GVHD grades II-IV if treated in LAF rooms compared to conventional isolation rooms (35).

Nutrition and oral intake, that keeps the gastrointestinal tract functioning, may reduce the risk of gut inflammation, cytokine release, and prevent GVHD (36–38). In an early study, oral nutrition appeared to decrease the risk of acute GVHD grades II-IV in home care patients (16). However, with more patients, oral nutrition was no longer a significant factor, and the number of days spent at home became more important for reducing acute GVHD grades II-IV (17).

The type of antibiotics used also affects the gastrointestinal microbiota and may influence the risk of GVHD (39). Patients treated at home often received gentamicin for fever of unknown origin because it can be given once daily. In contrast, patients in the hospital received imipenem intravenously four times per day. This routine may have affected the gut microbiota in favor of less GVHD grades II-IV in the home care patients.

There is a correlation between moderate to severe acute GVHD and TRM (40, 41). Therefore, it can be expected that a lower incidence of moderate to severe acute GVHD would lead to improved TRM and survival (15). Other factors that may contribute to the development of acute GVHD grades II-IV primarily in the hospital include worse sleep, more stress, and less exercise. We improved the nutrition of the patients treated in the hospital by creating a special team of nurses who encouraged them to eat more and better food instead of relying on TPN. We also had a special kitchen for relatives staying in the hospital with the patients, allowing them to prepare special food that the patients liked.

Additionally, a physical therapist designed exercise programs for the patients, and they were allowed to take walks outside the hospital in the evening when the corridors were not crowded. All these improvements in hospital care were introduced and encouraged to mimic home care as much as possible. These measures may have also reduced the risk of acute GVHD among hospital patients in more recent years. Another reason for a more similar risk of acute GVHD between home care and hospital care patients may be that home care patients have spent significantly less time at home in recent years. In the early era, patients spent a median of 15 days at home during neutropenia, which was significantly longer than a median of 10 days more recently (15, 17, 21).

In the first era, one experienced physician and two experienced nurses cared for all home care patients. When home care was found to be safe, more nurses and physicians were involved. When home care patients came to the ward due to fever of unknown origin, the less experienced doctors were hesitant to send them home after instituting antibiotics. They were more likely to consult infectious disease specialists, who would recommend imipenem to cover pseudomonas aeruginosa. Indeed, the use of antibiotics like imipenem affects the gut microbiota and increases the risk of acute GVHD (39). However, imipenem is given IV four times daily, which precludes home care. The nurse generally goes to the patient’s home once but no more than twice a day.

That home care patients in more recent years had a similar probability of grades II-IV acute GVHD (44%) compared to hospital care patients (37%) may only be explained by fewer days spent at home, as tissue typing, HLA matching, donor type, immunosuppression, patient selection, and GVHD treatment were the same in both groups. The number of days spent at home was significant in preventing moderate to severe acute GVHD (17). Furthermore, the probability of grades II-IV acute GVHD among hospital patients remained unchanged at 32% and 37% in the two time periods, respectively.

It can be discussed whether a special team of doctors and nurses should care for home care patients. It seems advantageous to have the same team caring for all patients, not least so that patients have the same staff throughout the entire HCT journey. Training staff to understand the importance of patients staying at home may be crucial. That home care, with less acute GVHD grades II-IV and shorter time to discharge, was less expensive than hospital care is expected (15). Furthermore, severe complications like GVHD are costly due to long hospital stays and expensive treatments (42, 43). In addition, outpatient Auto-HCT and Allo-HCT was also less expensive than hospital isolation, as were reported in several studies (44–47).

It was debated whether the favorable outcomes in home care patients compared to hospital-treated patients were due to selection bias. We do not believe this is the case because the two groups were very well balanced for treatment characteristics such as donor type, HLA compatibility, immunosuppression, age, and conditioning (15, 17). Furthermore, there is no way to select patients expected to develop acute GVHD grades II-IV, apart from obvious risk factors like HLA match, age, and female donors to male recipients (48–50).

A study comparing cytokine levels in home care and hospital care patients shed some light on the finding that home care patients had less acute GVHD grades II-IV (21). Home care patients had lower levels of GM-CSF compared to hospital controls (p<0.007, **Table 2**). In that study, patients with low GM-CSF levels had improved survival (p=0.036). In experimental models, GM-CSF increased GVHD (51), and GM-CSF-licensed myeloid cells induced GVHD (52).

Furthermore, IFN-γ and IL-2 were shown to induce acute GVHD grades II-IV (53–55). Home care patients had lower levels of IFN-γ compared to hospital controls (p=0.007, **Table 2**) and a trend towards lower IL-2 (p=0.07). There was also a trend for better survival in hospital controls with lower-than-median levels of IFN-γ (21). Stress was reported to increase IFN-γ levels, and hospital care may induce more stress than staying at home (56).

Both, IL-13 and IL-5 were lowered in home care patients (**Table 2**). IL-13 induces IgE, has anti-inflammatory properties, and downregulates macrophage activity (57). We found that IgE levels were dramatically increased during acute GVHD grades II-IV (58). The low levels of IL-13 align with low acute GVHD grades II-IV in home care patients. High levels of IL-5 were reported in patients with acute GVHD grades II-IV and fit with the low levels in home care patients (55) (**Table 2**).

Some studies reported a lower incidence of acute GVHD with RIC as opposed to MAC, due to less damage caused by chemoradiotherapy that triggers acute GVHD (59, 60). If RIC patients were analyzed separately, home care patients had higher VEGF levels (p=0.001) and lower G-CSF levels (p=0.04) compared to hospital-treated patients (21). High VEGF levels were associated with less acute GVHD grades II-IV (61, 62). In contrast, G-CSF activates T-cells and induces acute GVHD (63, 64).

The low levels of several inflammatory cytokines, especially GM-CSF and IFN-γ, but also to some extent IL-13, IL-5, and possibly IL-2, associated with high levels of VEGF suggest that several mechanisms may be involved in the decreased risk of acute GVHD grades II-IV in home care patients compared to those in hospital isolation.

There are several advantages to home care, like less acute GVHD grades II-IV, lower TRM, and reduced costs. More US centers, such as colleagues at Duke, have started home care programs, including Allo-HCT and Auto-HCT (65). A prospective randomized study comparing home care and hospital care is underway to prove the superiority of home care over hospital isolation.

## 4 Conclusions

In conclusion, our two decades of experience with home care at Karolinska Hospital and a confirmatory study from Barcelona suggest, that following Allo-HCT, it seems safer to be treated at home than in the hospital. In the Karolinska setting, home care after HCT was found to be safe in adults and children, and it reduced the risk of acute GVHD, decreased transplant-related mortality, improved survival, and led to significantly lower levels of systemic proinflammatory cytokines compared to hospital-treated controls (**Figure 1**).

## Author contributions

OR: Conceptualization, Data curation, Formal analysis, Funding acquisition, Investigation, Methodology, Project administration, Resources, Software, Supervision, Validation, Visualization, Writing – original draft, Writing – review & editing. B-MS: Conceptualization, Data curation, Formal analysis, Investigation, Methodology, Project administration, Resources, Writing – original draft, Writing – review & editing. GM: Conceptualization, Data curation, Validation, Writing – review & editing, Methodology, Software, Visualization. BS: Conceptualization, Data curation, Funding acquisition, Investigation, Supervision, Validation, Writing – original draft, Writing – review & editing.
